# Applications of bio-enhanced cementitious composites for sustainable building materials: emission-reduction and carbon-sequestration pathways

**DOI:** 10.1007/s43393-026-00556-3

**Published:** 2026-10-06

**Authors:** Shrameeta Shinde, Charandatta Muddana, J. Jeffrey Morris, Yinjie J. Tang, Muhammad M. Sherif

**Affiliations:** 1 Department of Energy, Environmental and Chemical Engineering (EECE), Washington University in St. Louis, St. Louis, MO 63130 USA; 2 Department of Biology, College of Arts and Sciences, University of Alabama at Birmingham, Birmingham, AL 35205 USA; 3 Department of Civil, Construction, and Environmental Engineering, University of Alabama at Birmingham, Birmingham, AL 35294 USA

**Keywords:** Biomanufacturing, Bio cement, Carbon neutral, Microbial induced calcite precipitation, Knowledge graph, Microbial metabolism

## Abstract

This article uses knowledge mining to examine microbial strengthening mechanism in cementitious composites. Knowledge graphs and large language models (LLMs) support literature collection and curation to link established findings with recent advances in the development of bio-cement. The review covers pathways of microbially induced calcite precipitation (MICP), microbial species, reinforcement/delivery strategies, metabolism under cementitious environments, and functional properties (i.e., self-healing, and carbon sequestration). The review article discusses the microbial physiology within cementitious matrices that consist of high alkalinity, restricted mass transport, and biomineralization. The article examines engineering limitations, including multistage and relatively slow production, dependence on moisture and nutrients, treatment byproducts, nonuniform precipitation, and uncertain long-term viability. Priorities include standardized field testing, protective delivery systems, omics-based analysis of microbial responses in cement, and machine learning ready databases. Coupling transcriptomics, metabolomics, metabolic flux analysis, and genome-scale models with AI/ML or digital twins may support strain selection and prediction of mineralization and self-healing. However, these approaches require interoperable datasets and field validation. The paper elaborates that bio-enhanced cementitious composites can support emission reduction and durable carbon sequestration. Whereas net negative performance must be demonstrated through complete life cycle analysis that accounts for greenhouse gases and microbial survivability in cementitious environments.

## Introduction

The construction sector is a major contributor to global greenhouse gas emissions, primarily due to the extensive use of conventional cementitious materials. Ordinary Portland cement (OPC) production accounts for approximately 7%–8% of anthropogenic CO_2_ emissions, driven by energy-intensive processing and limestone calcination (Andrew 2019; Habert et al. 2020; Scrivener et al. 2018). This significant environmental impact has accelerated the search for sustainable and low-carbon alternatives. In recent years, researchers have explored the development of bio-based composites with superior properties (Amorim et al. 2026; Azidhak et al. 2026; Grandgeorge et al. 2024; Liao et al. 2026; Lin et al. 2026). Consequently, increasing attention has been directed toward bio-based and bio-enhanced materials that can simultaneously reduce environmental footprints and enhance the performance and durability of construction systems (Castro-Alonso et al. 2019; De Belie et al. 2018; Lin et al. 2023a, b). Bio-enhanced cementitious composites have emerged as a promising class of materials incorporating biological agents such as bacteria, enzymes, and bio-derived additives into cement matrices. Foundational studies established microbial-induced calcium carbonate precipitation (MICP) as a viable mechanism for improving material properties and enabling autonomous crack healing (De Muynck et al. 2010; Jonkers et al. 2010). In this process, microorganisms precipitate CaCO_3_, which fills microcracks, reduces permeability, and enhances durability. These early investigations laid the groundwork for bio-concrete as a sustainable alternative to conventional repair strategies (Achal et al. 2010; Wiktor and Jonkers 2011).

Between 2015 and 2022, research significantly advanced the application of MICP and related biotechnologies, demonstrating improvements in compressive strength, crack-healing efficiency, and resistance to environmental degradation (Achal and Mukherjee 2015; Seifan et al. 2016; Sun et al. 2021; Wang et al. 2014). Bacteria-based self-healing systems have shown effective crack sealing under diverse conditions, confirming their practical feasibility (Elgendy et al. 2024; Wang et al. 2012a, b). Additionally, MICP was successfully applied in geotechnical engineering for soil stabilization, highlighting its broader applicability in sustainable construction (Cheng et al. 2013; DeJong et al. 2011). Recent developments (2023–2026) have focused on enhancing multifunctionality and carbon sequestration potential. Bio-enhanced cementitious composites are increasingly recognized for their ability to act as carbon sinks through biomineralization and the incorporation of carbon-rich materials such as biochar (Li et al. 2024; Zaid et al. 2024). Advances in engineered living materials and optimized microbial systems have further improved performance and durability (Jones et al. 2022; Qian et al. 2010), positioning these composites as key enablers of carbon-negative construction and circular material systems (Ahmed et al. 2024; Wang and Wang 2025). Accordingly, this study explores the role of MICP in production of bio-enhanced cementitious composites and their potential to transform the construction industry toward a sustainable and carbon-conscious future.

**Fig. 1:**
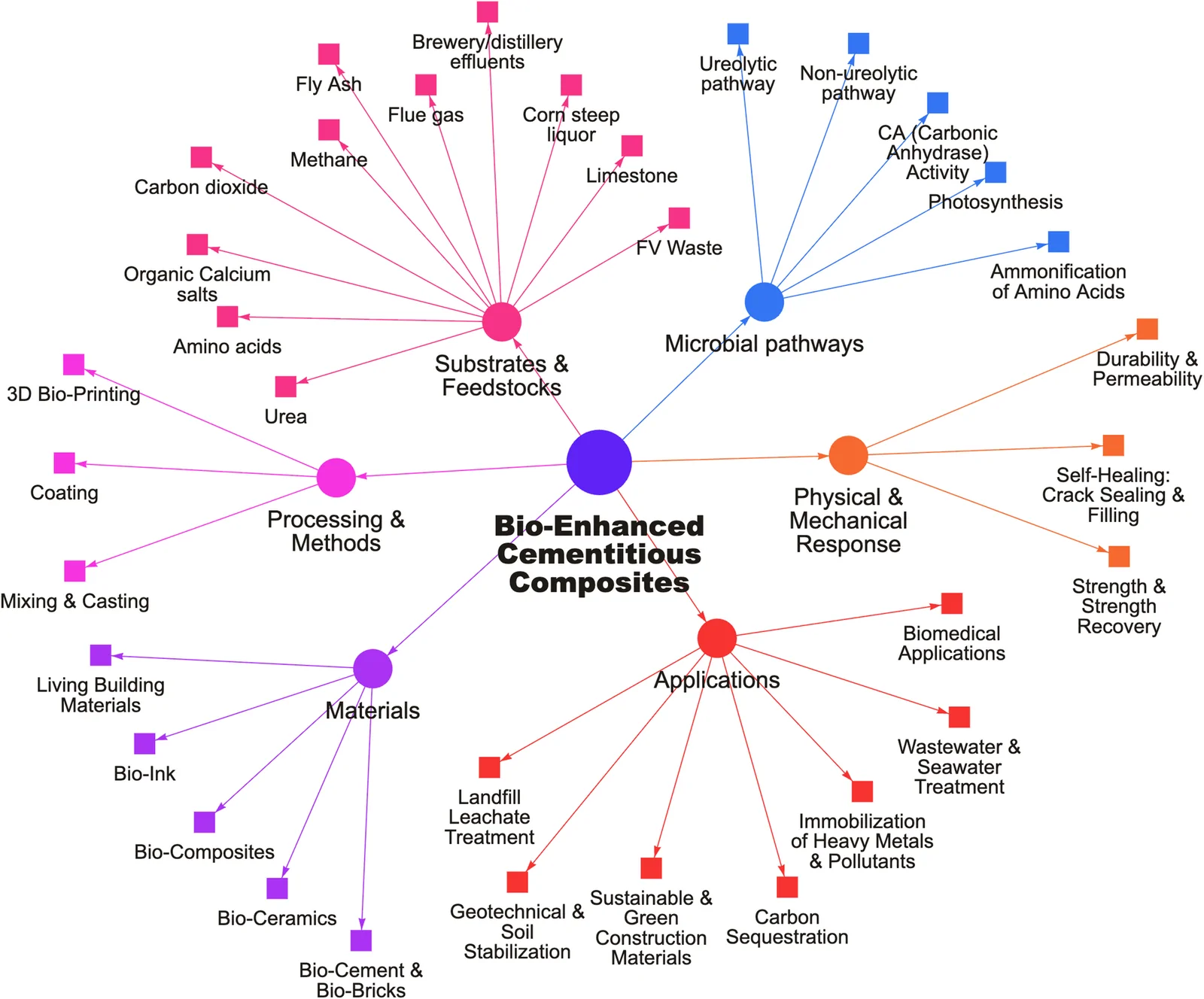
Transforming Data into Insight: Knowledge Graphs (KG) powered by LLMs. This KG illustrates major research clusters in bio-cementitious composites, with nodes representing the most representative keywords associated with each cluster. The graph was constructed using NEKO by analyzing abstracts from 200 research articles on bio-cementitious materials indexed in PubMed. It provides a comprehensive, integrative overview of bio-cement production pathways, encompassing the range of substrates utilized, the resulting mechanical properties and material characteristics, and their translation into practical, real-world applications

To provide a comprehensive overview of recent advances in interdisciplinary bio-cement research, we employed the NEKO (Network for Knowledge Organization) pipeline to systematically construct a literature-derived knowledge graph, enabling the mapping and analysis of the bio-cementitious composites research landscape (Xiao et al. 2025). Several researchers used an LLM-generated knowledge graph to survey the biotechnology literature (Lima et al. 2024; Muddana et al. 2026). In this review paper, NEKO was used to develop a navigation tool to map the research directions and associated applications. From over 200 PubMed abstracts, GPT (OpenAI) extracted key concepts (e.g., “microbial pathways,” “substrates”) and their relationships, while Sentence-Transformers and Word2Vec linked semantically related terms. In this graph, publications are nodes and edges encode content similarity between their extracted entities, enabling targeted exploration of themes and rapid identification of relevant work. Using PyVis, the network was visualized and clustered into interpretable research domains (Fig. 1). The figure was used to rapidly identify the thematic structure of the bio-cementation. Each cluster was then surveyed to identify the associated literature, and the final reference set was assembled through expert selection focused on the most current and impactful advances within those clusters. As a result, the novelty of this review paper lies in its comprehensive synthesis and linkage of microbial species, MICP and enzyme induced carbonate precitipation (EICP) pathways, immobilization strategies, and emerging carbon-negative applications of bio-enhanced cementitious composites. A Knowledge Graph (KG) + LLM workflow is well suited to writing a review paper, particularly on multidisciplinary topics where the central challenge is connecting materials, organisms, mechanisms, processes, and applications that are scattered across separate literatures. The KG makes those connections explicit and traceable, while the LLM helps navigate and synthesize them. In practice, the workflow has three stages: (1) define the review's scope and the specific questions it should answer; (2) build a literature-derived Knowledge Graph that captures the key entities and the relationships between them; and (3) interrogate the KG through a combination of LLM-assisted querying and expert judgment to surface patterns, gaps, and cross-domain links that inform the review's structure and arguments.

## Distinct contribution of this review

Recent reviews have summarized key aspects of bio-cementation, including microbial self-healing cementitious composites, fungal biomineralization, non-ureolytic carbonate precipitation, bacterial optimization, MICP engineering applications, and carbon-negative construction materials (Table 1). However, previous reviews generally focus on specific pathways, organisms, or applications. In contrast, this review presents an end-to-end pathway-to-deployment framework for systematically evaluating bio-enhanced cementitious systems based on six criteria: (1) Carbon source, calcium source, and biological or enzymatic pathway; (2) Precipitation kinetics, mineral identity, and reaction byproducts; (3) Microbial delivery, immobilization, and survivability; (4) Compatibility with cementitious binders and pore transporters; (5) Recovery of mechanical, transport, and durability properties; and (6) Environmental impacts and life-cycle carbon balance.

**Table 1:** Selected summary of MICP review papers

Representative review	Organized lens & principal strength	Boundary of the review	Added value supplied by the present review
Iqbal et al. (2021)	Bio-cementation in construction materials; broad overview of organisms, processes, and applications	A common end-to-end route-to-deployment test and an explicit net-carbon gate are not its organizing core	Compares ten route families through feedstock, byproducts, delivery, matrix compatibility, performance, carbon, and maturity
Van Wylick et al. (2021)	Filamentous fungi for concrete self-healing; deep fungal biology and candidate-organism perspective	Intentionally organism- and application specific	Places fungal biomineralization beside bacterial, photosynthetic, and enzyme routes across several applications using the same criteria
Jain et al. (2021)	Denitrification-based carbonate precipitation; deep non-ureolytic mechanism and building-material potential	Single-pathway focus	Compares denitrification with ureolysis, sulfate reduction, methane and iron routes, photosynthesis, FICP, and EICP, including byproducts and exposure
Zhang et al. (2023)	MICP fundamentals and engineering applications; strong discussion of field feasibility across sectors	Closest broad engineering comparator; it does not combine an AI semantic map with an explicit three-gate test of biological, engineering, and climate claims	Adds cementitious-composite integration, a carbon-claim gate, and graph-informed identification of cross-domain evidence gaps
Wong et al. (2024)	Microbial self-healing concrete; microbiology, survival, environmental variables, longevity, and scale-up	Self-healing concrete is the central application	Applies the biological-engineering-climate gates to several material functions and construction sectors
Zhu et al. (2024)	Bacterial improvement schemes; efficiency, cultivation, preservation, and cost	Bacterial optimization is the central unit of analysis	Connects optimization to mass transport, byproduct control, binder compatibility, life-cycle burden, and readiness
Kharissova et al. (2024)	Carbon-negative footprint materials across many material classes; definitions and broad carbon-capture landscape	Not specific to bio-cementitious pathways, structural performance, or MICP delivery	Tests carbon-negativity claim specifically for biomineralized cementitious systems using a declared system boundary and durable-removal criterion

The review of the ten biomineralization pathways, four delivery strategies, and six construction applications distinguishes three often-conflated concepts: biological feasibility, engineering feasibility, and climate credibility. Calcium carbonate precipitation demonstrates biological activity but does not necessarily ensure effective delivery, structural recovery, durability, scalability, or net-negative carbon performance. Similarly, rapid precipitation may improve crack sealing but can be constrained by ammonium generation, high reagent demand, clogging, and life-cycle emissions, whereas slower pathways may better align with the cementitious matrix, service environment, and sustainability objectives. This review also identifies where direct comparisons remain scientifically unreliable because studies report urease activity, microbial concentration, calcium carbonate yield, crack closure, curing conditions, and durability recovery using inconsistent units and test procedures. Rather than treating published results as directly comparable, the proposed framework distinguishes reported evidence from inferred performance and identifies the key information needed for meaningful future comparisons. The knowledge graph (Fig. 1) complements this framework by highlighting cross-domain relationships without replacing critical evaluation of literature. Together, these contributions provide a decision-support framework for selecting pathways, delivery strategies, and research priorities for practical bio-enhanced cementitious systems.

## MICP pathways

Calcite is precipitated using chemical reactions via carbon dioxide hydrolysis. Atmospheric (gas) carbon dioxide dissolves in aqueous form as it contacts liquid water until an equilibrium is achieved (Eq. 1). Through carbon dioxide hydrolysis, CO_2_ reacts with water and generates carbonic acid (Eq. 2). H_2_CO_3_ is diprotic and donates one proton to water to form bicarbonate (${\mathrm{HCO}}_{3}^{ - }$) and hydronium (H_3_O^+^) as described by Eq. 3. The bicarbonate can donate a second proton to form carbonate (${\mathrm{CO}}_{3}^{2 - }$) and another hydronium (H_3_O^+^) as described by Eq. 4. In the presence of calcium ions (Ca^2^⁺) and under alkaline conditions, calcite is formed as shown in Eq. 5. However, the equilibrium of Eq. 2 lies strongly towards the left, with a small fraction of dissolved CO_2_ present as carbonic acid. Also, the hydronium reduces the pH of the aqueous solutions. As a result, the calcite yield is limited (Hazarika and Yadav 2023). MICP serves as an alternative to purely chemical pathways.

1 $$ {\mathrm{CO}}_{2} \left( g \right) \leftrightarrow {\mathrm{CO}}_{2} \left( {aq} \right) $$

2 $$ {\mathrm{CO}}_{2} \left( {aq} \right) + {\mathrm{H}}_{2} {\mathrm{O}}\left( l \right) \to {\mathrm{H}}_{2} {\mathrm{CO}}_{3} \left( {aq} \right) $$

3 $$ {\mathrm{H}}_{2} {\mathrm{CO}}_{3} \left( {aq} \right) + {\mathrm{H}}_{2} {\mathrm{O}}\left( l \right) \leftrightarrow {\mathrm{HCO}}_{3}^{ - } \left( {aq} \right) + {\mathrm{H}}_{3} {\mathrm{O}}^{ + } \left( {aq} \right) $$

4 $$ {\mathrm{HCO}}_{3}^{ - } \left( {aq} \right) + {\mathrm{H}}_{2} {\mathrm{O}}\left( l \right) \leftrightarrow {\mathrm{CO}}_{3}^{2 - } \left( {aq} \right) + {\mathrm{H}}_{3} {\mathrm{O}}^{ + } \left( {aq} \right) $$

5 $$ {\mathrm{Ca}}^{2 + } + {\mathrm{CO}}_{3}^{2 - } \leftrightarrow {\mathrm{CaCO}}_{3} $$

The principle behind MICP is the ability of microorganisms to increase pH and dissolved inorganic carbon (DIC), creating supersaturation conditions for CaCO₃ precipitation in the presence of calcium ions (Ca^2^⁺) (Zhu et al. 2024). Bacterial cell surfaces, often carrying a net negative charge, attract Ca^2^⁺ ions, creating zones of high calcium concentration where calcite precipitates (Rodriguez-Navarro et al. 2003). The precipitated calcium carbonate forms different polymorphs, primarily calcite, which is preferred for its stability and strong inter-particle bonds (Dhami et al. 2013). The specific polymorph form depends on microbial species, environmental chemical composition, calcium source, temperature, and pH (De Muynck et al. 2010; Stocks-Fischer et al. 1999).

**Fig. 2:**
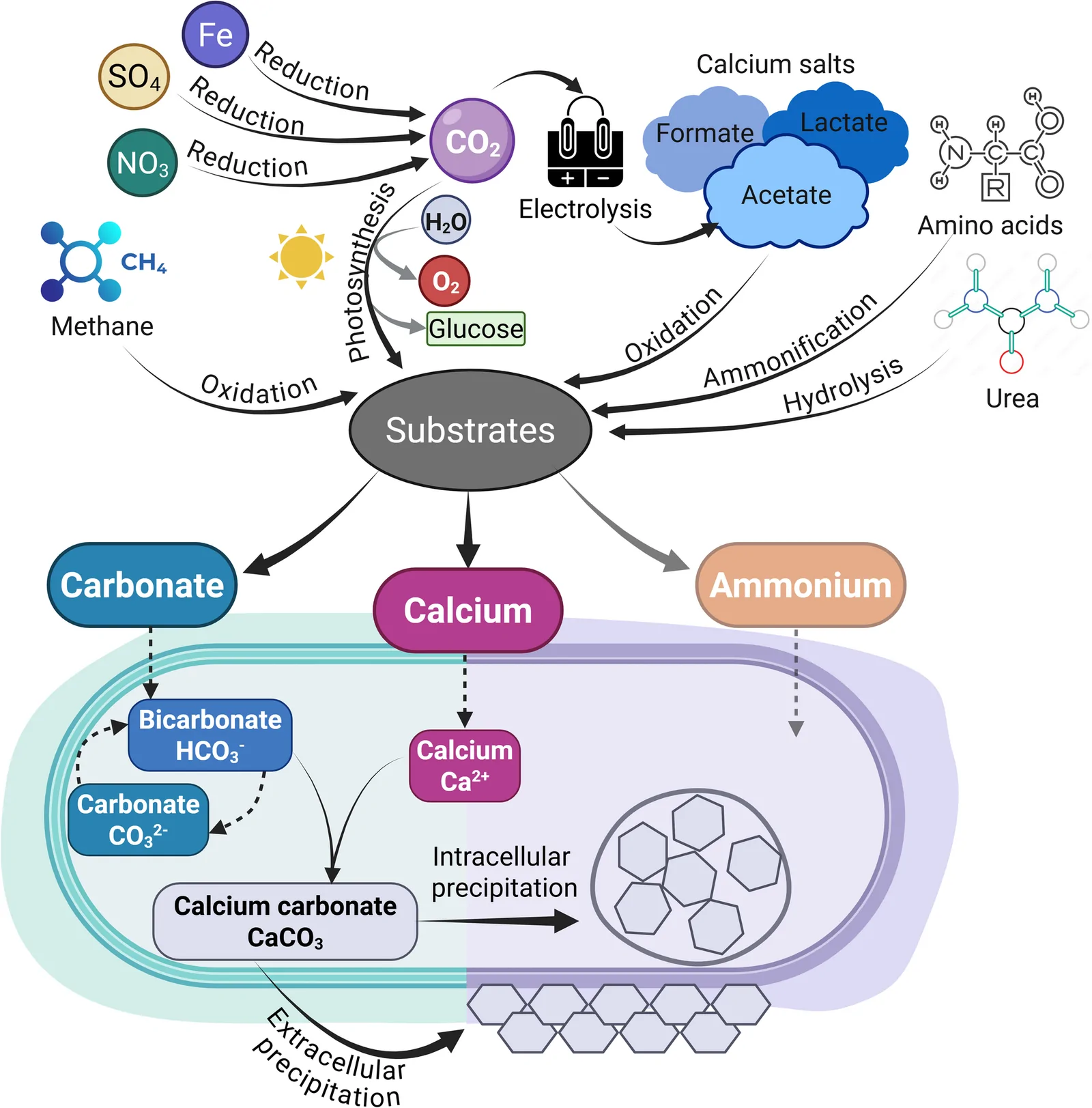
Multi-Substrate-Driven MICP. Microbially driven calcium carbonate precipitation utilizes multiple carbon substrates, including carbon–neutral sources such as CO_2_ and methane, under aerobic and anoxic conditions. Microbial metabolism of these substrates alters pH and dissolved inorganic carbon, facilitating CaCO_3_ precipitation. Created in https://BioRender.com

Microorganisms exhibit biochemical versatility, as depicted in Fig. 1, in mediating carbonate precipitation, presenting both challenges and opportunities for engineering optimization. The ureolytic pathway is the most efficient but raises environmental concerns due to its ammonia byproduct. Consequently, research is focusing on non-ureolytic alternatives, particularly those using organic salts or involving CO_2_ sequestration via carbonic anhydrase, to achieve a balance between efficiency and environmental sustainability (Fahimizadeh et al. 2022; Hazarika and Yadav 2023; Hemayati et al. 2023). Another strategy being explored is extracellular polymeric substances (EPS)—mediated precipitation. EPS functions as high-molecular-weight templates that drive passive biomineralization by sequestering Ca^2^⁺ through specialized functional groups and utilizing electrostatic cell-surface attractions, creating localized supersaturation (Dhami et al. 2013). Research on *Curvibacter* sp., *Arthrobacter* sp., and *Bacillus licheniformis* confirms that the specific protein and carbohydrate composition of these biopolymers strictly governs the nucleation, morphology, and polymorphism of the resulting mineral phases (Tourney and Ngwenya 2009; Zhang et al. 2021). The following sections provide a description of various microbial pathways for precipitating calcites along with a pictorial representation (Fig. 2). Also, Table 1 summarizes the major biological MICP pathways with a comparison of precipitation efficiency, byproducts, environmental compatibility, advantages, limitations and suitability. The precipitation-efficiency descriptors in Table 2 (very high, high, moderate-high, and moderate) are qualitative, relative classifications synthesized from the cited literature using the reported precipitation rate, carbonate yield, and consistency under each study's conditions. Because protocols, units, substrates, and curing conditions differ among studies, these descriptors are not based on universal quantitative thresholds and should not be interpreted as direct cross-study rankings.

**Table 2:** Summary of advantages, limitations, and applications of most discussed MICP pathways

Pathway	Precipitation efficiency	Byproducts	Environmental compatibility	Advantages	Limitations	Applications	Representative references
Ureolytic	Very High	${\mathrm{NH}}_{4}^{ + }$and${\mathrm{NH}}_{3}$	Moderate	Rapid precipitation; high cementation efficiency; extensively investigated; easy process control	Ammonia generation; requires bacterial cultivation; dependent on nutrients	Soil stabilization; crack healing; bio-cementation; erosion control; repair of cement-based structures	DeJong et al. (2010), Dhami et al. (2013), Stocks-Fischer et al. (1999)
Denitrification	High	${\mathrm{N}}_{2}$and Traces of${\mathrm{N}}_{2} {\mathrm{O}}$if an incomplete reaction	High	No ammonia production; suitable for anaerobic environments; simultaneous nitrate removal	Slower than ureolysis; requires nitrate and organic electron donor	Ground improvement; groundwater remediation; subsurface bio-cementation	Hammes et al. (2003), van Paassen et al. (2010a, b)
Sulfate-Reduction	Moderate	${\mathrm{H}}_{2} {\mathrm{S}}$and Sulfides	Moderate	Suitable for anaerobic environments; immobilizes heavy metals	Sulfide toxicity; odor; corrosion; relatively slow kinetics/reaction	Mine tailings; contaminated sediments; immobilization of metals	Enning and Garrelfs (2014)
Methane-Oxidation	Moderate	${\mathrm{CO}}_{2}$and Biomass	High	Simultaneous methane mitigation and biomineralization	Slow microbial growth; limited engineering-scale validation	Carbon sequestration landfill covers; environmental remediation	Boetius et al. (2000), Ganendra et al. (2014), Zhu and Dittrich (2016a)
Photosynthesis	Moderate-High (light dependent)	${\mathrm{O}}_{2}$	Very High	Carbon sequestration; sustainable; environmentally benign	Requires light; not suitable for subsurface applications	Surface bio-cementation; restoration; marine structures; carbon capture	Benzerara et al. (2014), Brininger et al. (2025), Jiang et al. (2013)
Enzymatic	High	${\mathrm{NH}}_{4}^{ + }$and${\mathrm{NH}}_{3}$	Moderate	Eliminates bacterial cultivation; effective in fine-grained soils; lower bio-clogging risk; easier process control	Cost of enzymes; limited enzymatic stability; generation of ammonia	Soil stabilization; crack repair; dust suppression; sand consolidation; heritage conservation	Gowthaman et al. (2021), Neupane et al. (2013)

### Ureolytic pathway

The ureolytic pathway (Eqs. 6–9) is the most studied and widely used mechanism for inducing calcite precipitation (Hazarika and Yadav 2023). This is due to its simplicity, ease of control, rapid reaction rates, and ability to generate substantial calcite promptly (DeJong et al. 2006). The microbial strains hydrolyze urea by urease in the cytoplasm, producing carbonic acid and ammonia. The ammonia dissolves, forming ammonium ions and releasing hydroxide ions. The hydroxide ions increase the pH resulting in a shift in the equilibrium towards producing carbonate ions. The intercellular carbonate ions react with the bound calcium ions, leading to the precipitation of calcium carbonate (CaCO_3_) (DeJong et al. 2010; Okwadha and Li 2010). It is worth noting that the release of ammonia (DeJong et al. 2010) causes leaching of calcium hydroxide [Ca(OH)_2_], corrosion of steel reinforcement and harsh environments for microbial survivability. As such, it reduces the durability of cementitious composites and limiting calcite precipitation.

6 $$ {\mathrm{CO}}\left( {{\mathrm{NH}}_{2} } \right)_{2} + {\mathrm{H}}_{2} {\mathrm{O}} \to {\mathrm{NH}}_{2} {\mathrm{COOH}} + {\mathrm{NH}}_{3} $$

7 $$ {\mathrm{NH}}_{2} {\mathrm{COOH}} + {\mathrm{H}}_{2} {\mathrm{O}} \to {\mathrm{H}}_{2} {\mathrm{CO}}_{3} + {\mathrm{NH}}_{3} $$

8 $$ {\mathrm{NH}}_{3} + {\mathrm{H}}_{2} {\mathrm{O}} \leftrightarrow {\mathrm{NH}}^{4 + } + {\mathrm{OH}}^{ - } $$

9 $$ 2{\mathrm{OH}}^{ - } + {\mathrm{H}}_{2} {\mathrm{CO}}_{3} \leftrightarrow {\mathrm{CO}}_{3}^{2 - } + 2{\mathrm{H}}_{2} {\mathrm{O}} $$

*Sporosarcina pasteurii* (*S. pasteurii*) is one of the most common bacterial strains for bio-cementation due to its high urease activity and resistance in alkaline and high-ammonia environments (Ma et al. 2020). Additionally, researchers have explored the utilization of filamentous fungi due to the formation of structural hyphal networks and bio-cementation through their urease activity (Van Wylick et al. 2021). Table 3 illustrates various strains that have been investigated for ureolytic MICP activity. Although ureolysis has demonstrated unconfined compressive strengths of up to 12.4 MPa in bio-cemented sand (Van Paassen et al. 2010a, b), its application is constrained by the production of 2 mol NH_4_⁺ per mol urea hydrolyzed, which poses environmental and treatment challenges (Stocks-Fischer et al. 1999). From a practical perspective, ureolytic MICP is more amenable to field implementation because it predominantly employs endospore-forming bacteria such as *S. pasteurii* and *Bacillus* spp., whose spores can be stored and transported as dry formulations with long shelf life and high environmental tolerance (Carter et al. 2023; De Muynck et al. 2010).

**Table 3:** Microbes for ureolytic pathway

Genus/Species	Strains	Properties
*Sporosarcina pasteurii*	DSM 33 (Han et al. 2021; Lapierre et al. 2020; Ma et al. 2020)	High urease activity (rapid urea hydrolysis); Thrive in alkaline conditions; Tolerant to high concentrations of ammonia & salts; Forms resistant spores; Enhanced urease activity & calcite production via mutations
	ATCC 11869 (Han et al. 2021; Lapierre et al. 2020; Ma et al. 2020)	
	BNCC 337394 (Han et al. 2021; Lapierre et al. 2020; Ma et al. 2020)	
	MTCC 1761 (Achal et al. 2009b)	
*Bacillus *Species	*B. sphaericus* (Wang et al. 2012a, b; Wang et al. 2014)	High urease activity; Diverse environmental tolerances; Bacterial spores used for healing concrete cracks; Genetically enriched microbe as a self-healing agent
	LMG 22557 (Wang et al. 2012a, b; Wang et al. 2014)	
	*B. megaterium *SS3* (*Dhami et al. 2013*), *MTCC 1684 and BSKAU (Krishnapriya et al. 2015)	
	*B. flexus *BSKNAU (Krishnapriya et al. 2015)	
	*B. licheniformis* (Krishnapriya et al. 2015)	
	*B. subtilis* (Dhami et al. 2013; Khaliq and Ehsan 2016; Sarkar et al. 2015)	
	*B. haynesii *SK1 (Shen et al. 2025)	
	*B. pseudofirmus *DSM 8715 (Jonkers et al. 2010)	
	*B. cohnii *DSM 6307 (Jonkers et al. 2010)	
	*B. cereus *SS5 (Dhami et al. 2013); CH-13 (Kang et al. 2014)	
	*B. thuringiensis* SS15 (Dhami et al. 2013); CH-21 (Kang et al. 2014)	
*Lysinibacillus* species	*L. fusiformis *5.1 (Leeprasert et al. 2022); SS18 (Dhami et al. 2013)	High urease activity; Efficient CaCO_3_ precipitation; Good spore viability; EPS production affecting calcite morphology
	CH-11 (Kang et al. 2014)	
	*L. sphaericus *CH-5 (Kang et al. 2014)	
	*L. xylanilyticus *4.3 (Leeprasert et al. 2022)	
Filamentous fungi	*Neurospora crassa* (Martuscelli et al. 2020)	Hyphae create nucleation networks; High cation-binding ability; Urease activity; Forms calcite in nutrient-poor, alkaline environments
	*Penicillium chrysogenum* (Martuscelli et al. 2020)	
	*Aspergillus nidulans (MAD1445)* (Luo et al. 2018)	
	*Fusarium oxysporum* (Luo et al. 2018)	
	*Trichoderma reesei* (Luo et al. 2018)	
	*Fusarium cerealis* (Zhao et al. 2022)	
	*Phoma herbarum* (Zhao et al. 2022)	
	*Mucor hiemalis* (Zhao et al. 2022)	
	*Alkaliphilic *Species (Jiang et al. 2017; Ozimek and Hanaka 2021)	
	*Oligotrophic *Species (Jiang et al. 2017; Ozimek and Hanaka 2021)	

### Aerobic oxidation

The aerobic oxidation of organic carbon is a pathway that can be utilized by heterotrophic bacteria to precipitate calcite. In this pathway, organic acids such as lactate, acetate, and formate, are used as a calcium source. Equations 10–12 illustrate the dissolution of calcium lactate, acetate, and formate, respectively. Equations 13–15 show the chemical reaction for the microbial aerobic oxidation of lactate, acetate, and formate. This aerobic oxidation produces carbon dioxide. The dissolution and hydration of carbon dioxide produce bicarbonate by Eq. 16 (Choi et al. 2017). Under alkaline conditions, the bicarbonate equilibrates to ${\mathrm{CO}}_{3}^{2 - }$ ions (Eq. 17), which then precipitate as ${\mathrm{CaCO}}_{3}$ in the presence of Ca^2^⁺ (Eq. 5). The aerobic oxidation pathway is a cleaner alternative under active research due to elimination of ammonia production (Hemayati et al. 2023). Table 4 illustrates the various strains that have been investigated for MICP through aerobic oxidation.

10 $$ {\mathrm{Ca}}\left( {{\mathrm{C}}_{3} {\mathrm{H}}_{5} {\mathrm{O}}_{3} } \right)_{2} \to {\mathrm{Ca}}^{2 + } + 2{\mathrm{C}}_{3} {\mathrm{H}}_{5} {\mathrm{O}}_{3}^{ - } $$

11 $$Ca\left( {{\mathrm{CH}}_{3} {\mathrm{COO}}} \right)_{2} \to {\mathrm{Ca}}^{2 + } + 2{\mathrm{CH}}_{3} {\mathrm{COO}}^{ - } $$

12 $$ {\mathrm{Ca}}\left( {{\mathrm{HCOO}}} \right)_{2} \to {\mathrm{Ca}}^{2 + } + 2{\mathrm{HCOO}}^{ - } $$

13 $${\mathrm{C}}_{3} {\mathrm{H}}_{6} {\mathrm{O}}_{3}^{ - } + 3{\mathrm{O}}_{2} \to 3{\mathrm{CO}}_{2} + 3{\mathrm{H}}_{2} {\mathrm{O}} $$

14 $${\mathrm{CH}}_{3} {\mathrm{COO}}^{ - } + 2{\mathrm{O}}_{2} \to 2{\mathrm{CO}}_{2} + {\mathrm{H}}_{2} {\mathrm{O}} + {\mathrm{OH}} ^{ - }$$

15 $$ 2{\mathrm{HCOO}}^{ - } + {\mathrm{O}}_{2} \to 2{\mathrm{CO}}_{2} + 2{\mathrm{OH}}^{ - } $$

16 $$ {\mathrm{CO}}_{2} + {\mathrm{H}}_{2} {\mathrm{O}} \leftrightarrow {\mathrm{HCO}}_{3}^{ - } + {\mathrm{H}}^{ + } $$

17 $${\mathrm{HCO}}_{3}^{ - } \leftrightarrow {\mathrm{CO}}_{3}^{2 - } + {\mathrm{H}} ^{ + }$$

**Table 4:** Microbes for aerobic oxidation of organic compounds

Genus	Strains
*Arthobacter*	*A. koreensis *CA15-8 (Shaheen et al. 2021)*; A. luteolus *LNR3 (Shaheen et al. 2021)
*Bacillus*	*B. subtilis* (Amieghemen et al. 2026); *B. safensis *MUGA 156 (Shaheen et al. 2021)*; B. pumilus *SH-B9 (Shaheen et al. 2021)*; B. australimaris *MCCC 1A05787 (Shaheen et al. 2021)*; B. idriensis* (Seidel et al. 2025)*; B. licheniformis* (Seidel et al. 2025)*; B. frigoritolerans* (Seidel et al. 2025)
*Brevibacillus*	*B. choshinensis* (Seidel et al. 2025)*; B. frigoritolerans* (Seidel et al. 2025)
*Chryseomicrobium*	*C. amylolyticum *JC16 (Shaheen et al. 2021)*; C. imtechense *HTHB4 (Shaheen et al. 2021)
*Corynebacterium*	*C. efficiens *strain YS-314 (Shaheen et al. 2021)
*Glutamicibacter*	*G. mysorens *LMG16219 (Shaheen et al. 2021)
*Metabacillus*	*Metabacillus dongyingensis* (Seidel et al. 2025)
*Planococcus*	*P. plakortidis *DSM 23997 (Shaheen et al. 2021)
*Rhodococcus*	*R. erythreus *S26 (Choi et al. 2021)*, R. opacus* (Szcześ et al. 2016)*, R. cerasti *and* R. fascians* (Lange-Enyedi et al. 2024)
*Solibacillus*	*S. silvestris*;* S. isronensis* (Seidel et al. 2025)
*Others*	*Lysinibacillus varians* (Seidel et al. 2025);* Peribacillus simplex* (Seidel et al. 2025)

Another process for MICP is ammonification of amino acids. Microorganisms degrade amino acids and release NH_3_ and CO_2_ as demonstrated by Eq. 18. The microbe *Myxococcus xanthus* hydrolyzes the NH_3_, increasing the pH and concentration of carbonate ions as described in Eq. 19 (Ben Chekroun et al. 2004; Rodriguez-Navarro et al. 2003). This results in favorable conditions for the precipitation of calcite as described earlier by Eqs. 16, 17, and 5 (Rodriguez-Navarro et al. 2003). It is worth noting that in marine environments, the enzyme carbonic anhydrase (CA) further accelerates the calcite precipitation, as the ammonification simultaneously consumes the hydrogen (H^+^) ions (Krause et al. 2018).

18 $$ Amino\;Acids + {\mathrm{O}}_{2} \to {\mathrm{NH}}_{3} + {\mathrm{CO}}_{2} + {\mathrm{H}}_{2} {\mathrm{O}} $$

19 $$ {\mathrm{NH}}_{3} + {\mathrm{H}}_{2} {\mathrm{O}} \to {\mathrm{NH}}_{4}^{ + } + {\mathrm{OH}}^{ - } $$

### Anoxic oxidation of organic carbon

In general, oxygen is the primary electron acceptor for organic carbon oxidation, as such, the absence of oxygen hinders the calcite precipitation. However, some microbial strains can use alternative electron acceptors, including nitrate reduction, sulfate reduction, iron reduction, and methanogenesis. The following sections present further details on the anoxic oxidations.

First, nitrate reduction is particularly favorable due to its high-standard free energy and can serve as a primary MICP pathway in the absence of oxygen. The denitrification process is illustrated by Eq. 20. The calcite precipitation processes are followed by Eqs. 16, 17, and 5. Due to the consumption of protons (H^+^) and production of bicarbonate (HCO_3_^−^) the processes increase alkalinity. This results in a high rate of calcite precipitation (Erşan et al. 2015; Pham et al. 2018). Also, denitrification is a promising alternative to ureolysis as it does not produce toxic ammonia and unpleasant odors (Tziviloglou et al. 2016). This process is observed in denitrifying bacteria like *Pseudomonas aeruginosa*, *Diaphorobacter nitroreducens* (Erşan et al. 2015) and *Castellaniella denitrificans* (van Paassen et al. 2010a, b). Under laboratory conditions, denitrification processes have slow carbonate precipitation compared to ureolysis at a rate of 0.3–0.5 g CaCO₃ L^⁻1^ d^⁻1^ (Erşan et al. 2015).

20 $$ 5{\mathrm{CH}}_{2} {\mathrm{O}} + 4{\mathrm{NO}}_{3}^{ - } + 4{\mathrm{H}}^{ + } \to 5{\mathrm{CO}}_{2} + 2{\mathrm{N}}_{2} + 7{\mathrm{H}}_{2} {\mathrm{O}} $$

Second, sulfate-reducing bacteria, such as *Desulfovibrio* sp. (Perito and Mastromei 2011) and *Desulfovibrionales* sp. (Braissant et al. 2007), precipitate calcium carbonate by using sulfate as an electron acceptor to produce HCO_3_^−^ and H_2_S as presented in Eq. 21. The bicarbonate increases alkalinity. The calcium carbonate is precipitated as illustrated by Eq. 22 in the presence of calcium ions (Ca^2+^) (Vasconcelos and McKenzie 1997). Sulfate-reducing MICP offers a low-carbon alternative by eliminating urea hydrolysis but is constrained by strict anaerobic conditions and hydrogen sulfide production (Enning and Garrelfs 2014; Mwandira et al. 2023). This process usually occurs after the depletion of oxygen and nitrogen. The process introduces toxicity in the form of sulfide and can react with metals such as iron as described in Eq. 23 resulting in a reduced durability (corrosion) for reinforced cementitious materials.

21 $$ 2{\mathrm{CH}}_{2} {\mathrm{O}} + {\mathrm{SO}}_{4}^{2 - } \to {\mathrm{H}}_{2} {\mathrm{S}} + 2{\mathrm{HCO}}_{3}^{ - } $$

22 $$ {\mathrm{Ca}}^{2 + } + 2{\mathrm{HCO}}_{3}^{ - } \to {\mathrm{CaCO}}_{3} + {\mathrm{CO}}_{2} + {\mathrm{H}}_{2} {\mathrm{O}} $$

23 $$ {\mathrm{Fe}}^{2 + } + {\mathrm{H}}_{2} {\mathrm{S}} \to {\mathrm{FeS}} $$

Third, methane-oxidizing bacteria (MOB) consume methane by reacting with electron acceptors (sulfate: ${\mathrm{SO}}_{4}^{2 - }$, nitrate: ${\mathrm{NO}}_{3}^{ - }$, iron: ${\mathrm{Fe}}^{3 + }$, and manganese: ${\mathrm{Mn}}^{4 + }$) and produce either bicarbonate (HCO_3_^−^) or carbon dioxide (CO_2_) as described in Eqs. 24–27. The calcium carbonate is precipitated as illustrated by Eqs. 16, 17 and 5 in the presence of calcium ions (Ca^2+^) (Boetius et al. 2000; Ganendra et al. 2014). In general, sulfate as an electron acceptor has the largest precipitation of calcite due to strong bicarbonate production. The nitrate pathway produces moderate calcite precipitation, while iron and manganese produce the smallest precipitation in calcite. This process is observed in bacterial species such as *Methylocystis parvus* and *Methylococcus trichosporium* (Ganendra et al. 2014).

24 $$ {\mathrm{CH}}_{4} + {\mathrm{SO}}_{4}^{2 - } \to {\mathrm{HCO}}_{3}^{ - } + {\mathrm{HS}}^{ - } + {\mathrm{H}}_{2} {\mathrm{O}} $$

25 $$ {\mathrm{CH}}_{4} + 4{\mathrm{NO}}_{3}^{ - } \to {\mathrm{CO}}_{2} + 4{\mathrm{NO}}_{2}^{ - } + 2{\mathrm{H}}_{2} {\mathrm{O}} $$

26 $$ {\mathrm{CH}}_{4} + 8{\mathrm{Fe}}\left( {{\mathrm{OH}}} \right)_{3} + 15{\mathrm{H}}^{ + } \to {\mathrm{HCO}}_{3}^{ - } + 8{\mathrm{Fe}}^{2 + } + 21{\mathrm{H}}_{2} {\mathrm{O}} $$

27 $$ {\mathrm{CH}}_{4} + 4{\mathrm{MnO}}_{2} + 7{\mathrm{H}}^{ + } \to {\mathrm{HCO}}_{3}^{ - } + 4{\mathrm{Mn}}^{2 + } + 5{\mathrm{H}}_{2} {\mathrm{O}} $$

Fourth, using carbon sources such as lactate (electron donor), microbes can reduce iron via extracellular electron transfer mechanisms as shown in Eq. 28. The reaction produces bicarbonate that can precipitate calcite in the presence of calcium ions (Ca^2+^) as discussed previously. However, this process is much less efficient due to production of protons (H^+^), which reduce the alkalinity and carbonate saturation. Moreover, the reduced ferrous iron (Fe^2+^) can compete with the calcium ions (Ca^2+^) to react with carbonate to form iron carbonate (FeCO_3_) minerals (Wang et al. 2023). Certain bacteria, including *Geobacter* spp.*, Shewanella* spp. and *Acidiphilium* sp. DSM 24941 (Sánchez-Róman et al. 2014), metabolize organic compounds such as acetate or lactate, producing bicarbonate (HCO_3_⁻) as a byproduct (Han et al. 2024). Additionally, an iron-reducing microbe *Orenia metallireducens* was shown to facilitate calcium-related carbonate precipitation by modulating pH through its metabolic activity, promoting the conversion of dissolved CO_2_ into bicarbonate and enhancing mineral formation under CO₂-rich conditions (Li et al. 2022).

28 $$ {\mathrm{C}}_{3} {\mathrm{H}}_{5} {\mathrm{O}}_{3}^{ - } + 4{\mathrm{Fe}}^{3 + } + 2{\mathrm{H}}_{2} {\mathrm{O}} \to 3{\mathrm{HCO}}_{3}^{ - } + 4{\mathrm{Fe}}^{2 + } + 5{\mathrm{H}}^{ + } $$

### Photosynthesis

Table 5 summarizes some of the microorganisms that utilize photosynthesis for MICP. Water with dissolved inorganic carbon and calcium reacts as illustrated by Eqs. 16 and 17. The calcite is deposited by calcium (${\mathrm{Ca}}^{2 + }$) and carbonate (${\mathrm{CO}}_{3}^{2 - }$) based on Eq. 5. However, at neutral pH most inorganic carbon is bicarbonate (${\mathrm{HCO}}_{3}^{ - }$). Photosynthetic microbes (cyanobacteria and microalgae) consume carbon dioxide. Inside microbes, CO_2_-concentrating mechanisms pump in ${\mathrm{HCO}}_{3}^{ - }$, convert it to CO_2_ in carboxysomes via carbonic anhydrase, and fix it with RuBisCO. The removal of carbon dioxide releases hydroxide and raises the pH. The increased pH drives the reaction towards the release of carbonate and increases the calcite precipitation. Applications for construction materials are limited due to the need for light and CO_2_ (Thompson and Ferris 1990). Cyanobacteria and microalgae have demonstrated CO_2_ fixation rates of approximately 0.2–2.0 g CO_2_ L^⁻1^ d^⁻1^ under optimized photobioreactor conditions, while outdoor cultivation systems typically achieve 10–40 g CO_2_ m^⁻2^ d^⁻1^ due to limitations from light availability, temperature variation, and mass transfer (Brennan and Owende 2010; Kumar et al. 2011). Photosynthetic and carbonic anhydrase-mediated MICP avoid ammonia emissions and facilitate CO_2_ sequestration; however, their limitations, enzyme stability, and higher production costs currently limit large-scale engineering applications (Achal and Mukherjee 2015; Zhu and Dittrich 2016a).

**Table 5:** Microbes for photosynthetic MICP

Genus	Strains
*Anabaena*	ATCC 33047 (Brininger et al. 2025)
*Chlorella*	*Chlorella* sp. (Ramanan et al. 2010)
*Rhodovulum*	*R. steppense *A-20, S-17–65 (Bundeleva et al. 2012)
*Spirulina*	*S. platensis* (Ramanan et al. 2010)
*Synechococcus*	PCC 8806 (Jansson and Northen 2010), PCC 7002 (Heveran et al. 2020), PCC 7942 (Martinho de Brito et al. 2022)
*Synechocystis*	PCC 6803 (Jiang et al. 2013)*, S. pevalekii *BDHKU 35101 (Sidhu et al. 2022)

Alternatively, lichen-induced carbonate precipitation (LICP) is a biomineralization process in which lichen symbioses (fungi, algae and/or cyanobacteria, and associated microorganisms) promote CaCO_3_ formation through coordinated metabolic activities. Carbonate precipitation is facilitated by photosynthetic CO_2_ fixation, fungal-mediated mineral weathering using secretion of organic acids like oxalic acid that releases Ca^2^⁺, EPS that provide nucleation sites, and, in some species, cyanobacterial nitrogen fixation that influences local alkalinity (Chen et al. 2000). These characteristics make lichens attractive for sustainable, potentially carbon-negative construction materials due to their ability to fix carbon, weather minerals, and form stable mineralized surfaces. However, their engineering application is currently constrained by slow growth, strong environmental dependence, limited process control, and insufficient evidence of significant improvements in the mechanical performance of cementitious materials (Krajanová 2023). Consequently, LICP is better regarded as a long-term, nature-inspired approach for carbon sequestration and surface mineralization rather than a practical technology for construction applications.

### Fungi induced carbonate precipitation (FICP)

Recent research has highlighted the potential of fungi to promote CaCO_3_ formation through urease activity, respiration, organic acid metabolism, and hyphal-mediated nucleation (De Muynck et al. 2010; Dhami et al. 2013). Fungal hyphae provide abundant nucleation sites for crystal growth and can penetrate microcracks and pores, enhancing mineral deposition. Compared with bacterial microbially induced carbonate precipitation (MICP), FICP offers advantages such as greater tolerance to alkaline environments, utilization of diverse carbon sources, and the potential for non-ureolytic carbonate precipitation, thereby reducing ammonium production (De Muynck et al. 2010). However, its application is limited by slower mineralization rates, fewer well-characterized fungal strains, and uncertainties regarding long-term performance in cementitious materials (Achal and Mukherjee 2015). Nevertheless, FICP is increasingly recognized as a complementary bio-cementation strategy for self-healing concrete, crack repair, soil stabilization, and sustainable construction materials (Dhami et al. 2013). Fungal isolates sourced from alkaline soils have shown urease activity and the capability to precipitate calcite (Devgon et al. 2024).

### Enzyme induced carbonate precipitation (EICP)

It is a cell-free method in which purified plant urease, such as jack bean urease, is combined with urea and Ca^2^⁺ to promote CaCO_3_ precipitation. As enzymes are significantly smaller than cells, EICP can effectively penetrate fine-grained soils and avoid issues related to microbial viability or contamination. However, the high cost of pure urease and the lack of biological control over nucleation are drawbacks. Research is ongoing to optimize enzyme kinetics, use waste-derived enzymes, and reduce ammonia byproducts (Nemati and Voordouw 2003). A further illustration of EICP involves employing engineered and purified carbonic anhydrase to capture atmospheric CO_2_ and convert it into bioavailable carbonate species. This enzyme, carbonic anhydrase (CA), catalyzes the reversible interconversion of CO_2_ and HCO_3_⁻, providing an effective and eco-friendly enzymatic pathway for carbon sequestration and removal (Hazarika and Yadav 2023).

## Parameters impacting MICP

The calcite precipitation rate of MICP is dependent on several factors, including microbial strains, substrates, soluble calcium sources, nutrients (feedstock), and environmental conditions (temperature and pressure). Microbial cultures can be harvested using bioaugmentation with high density lab-grown strains or bio-stimulation of native microbes (Iqbal et al. 2021; Zhu et al. 2024). In general, spore forming bacteria are preferred for engineering applications due to their resilience and ease of storage (Tuttle et al. 2025). Advancements in lyophilization have improved the shelf-life of spore forming bacteria significantly. The initial density of active bacterial cells influences the calcite precipitation rate. Although high microbial concentration can increase the calcite precipitation rate, it can reduce efficiency as nutrients are consumed rapidly and become scarce.

For ureolytic MICP pathways, chemically pure urea is the optimal substrate for urease. However, due to the high-manufacturing cost of reagent-grade urea, researchers have been searching for cost-effective alternatives such as urine and urea-rich wastewater (Yan et al. 2025). The calcium source influences the calcite crystal morphology (Raveh-Amit and Tsesarsky 2020). Calcium chloride is the most common calcium source. In recent years, cheaper waste materials like limestone powder, recycled concrete, and seashell powder have been investigated for calcium sources (Dawood Muhammad Iqbal et al. 2021). Nutrients (feedstock) are vital for microbial growth. Low-cost nutrients such as kitchen waste, chicken manure wastewater, and industrial byproducts have been utilized (Yan et al. 2025). Excessive concentration of urea and calcium sources can inhibit microbial activity due to osmotic stress (Arias et al. 2025) or ammonium toxicity (Lee et al. 2019). This can lead to rapid, uncontrolled precipitation and decreased efficiency (Konstantinou et al. 2021). Inhibitors such as heavy metals, acetohydroxamic acid, cementitious fillers (fibers, platelets, particles, and tubes) can limit the calcite precipitation (Lai et al. 2024; Omoregie et al. 2023, 2021; Wong et al. 2024). In contrast, most non-ureolytic microorganisms, including denitrifying, sulfate-reducing, and photosynthetic bacteria, require transport as metabolically active cultures under controlled environmental conditions, increasing logistical complexity and limiting scalability (Zhu and Dittrich 2016a).

Temperature and pH have an influence on microbial growth, enzyme reaction rate, and calcite precipitation (Lai et al. 2024; Omoregie et al. 2023, 2021; Wong et al. 2024). Microbially induced calcite precipitation is sensitive to medium pH leaning towards alkaline with an optimal range between 7.0 and 9.5. Fresh cementitious composites are strongly alkaline (pH 12 to 13). However, due to carbonation, environmental exposure (acid rain), leaching of alkaline components, and chemical/biological attack, the pH is slowly neutralized. In harsh conditions, the cementitious composites become acidic. Therefore, selecting an MICP pathway for construction applications should consider not only carbonate precipitation efficiency but also environmental impact, operational complexity, microbial preservation, transportation logistics, and field scalability (Achal and Mukherjee 2015; DeJong et al. 2010).

MICP is also impacted by the replacement of cement with supplementary cementitious materials (SCMs) such as slag, calcined clays, fly ash, and alkali activated materials. In general, SCMs modify pore solution chemistry, hydration products, pore structure, and calcium availability, which can influence bacterial viability, urease activity, and calcium carbonate precipitation. While high SCM replacement levels may reduce the availability of free calcium required for biomineralization, studies have shown that MICP remains effective in improving crack healing, reducing permeability, and enhancing durability when sufficient calcium is available (Achal et al. 2011; Chuo et al. 2020; De Muynck et al. 2010). Alkali-activated materials present formulation-specific challenges because calcium content, pore-solution chemistry, and alkalinity vary substantially with precursor, activator, and curing regime. A limited number of studies indicate that MICP can function in selected alkali-activated formulations when compatible calcium sources, alkali-tolerant microbial strains, and suitable curing conditions are used (Bundur et al. 2015; Chuo et al. 2020). These findings should not be generalized to alkali-activated materials as a class; performance must be demonstrated for each binder formulation. Glass pozzolans inhibit the activity of microbial strains (Amieghemen et al. 2026).

## Integration and immobilization of microbial strains

**Fig. 3:**
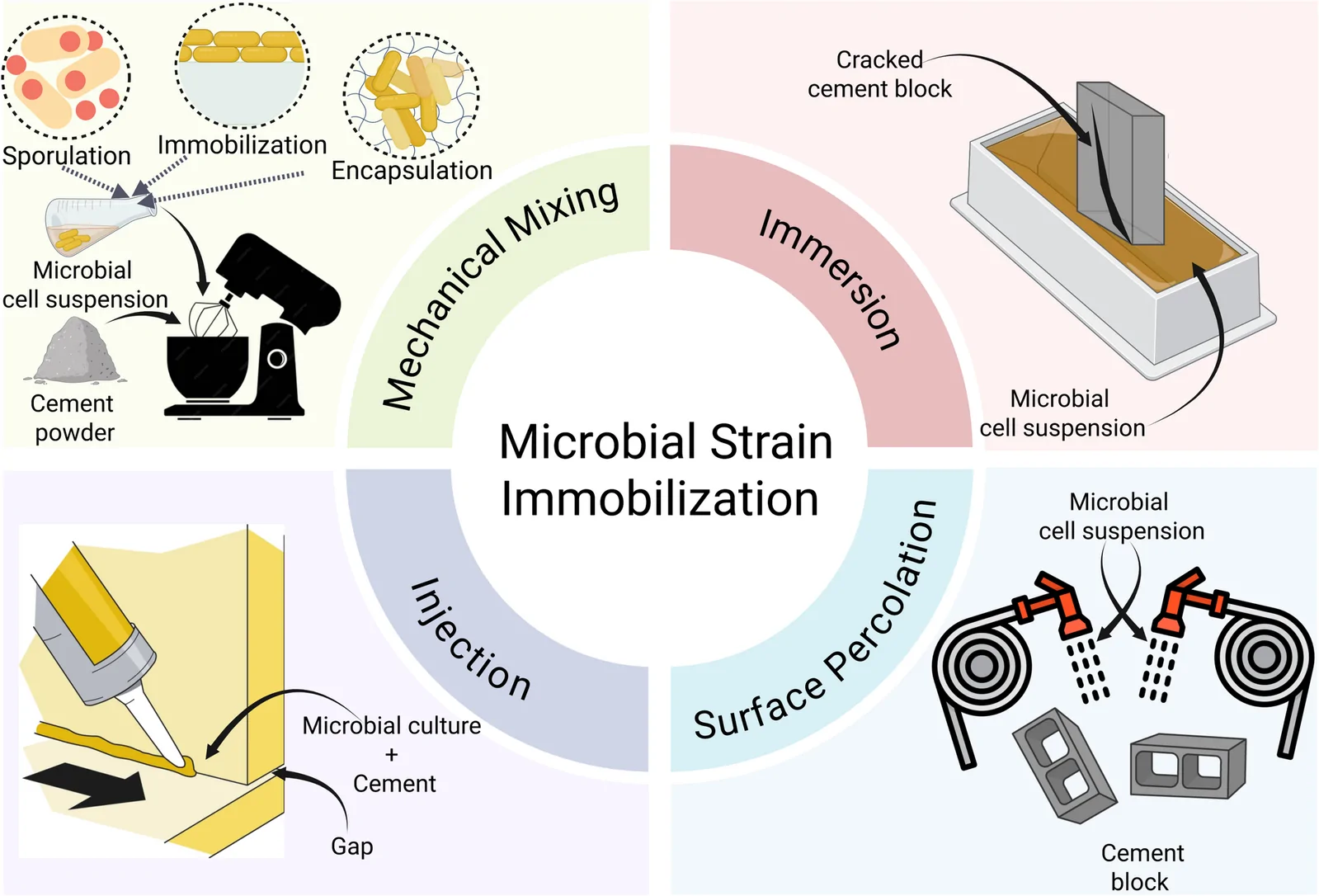
Integrated approaches to microbial strain immobilization. This figure illustrates the principal methodologies for microbial incorporation in bio-cementation processes, including mechanical mixing within the concrete matrix, direct injection into concrete with cracks/gaps, immersion of materials in microbial suspensions, and surface percolation techniques facilitating microbial transport through the porous network. Created in https://BioRender.com

Several techniques have been investigated to reinforce soil and cementitious composites with microbial strains (Fig. 3). Traditional cementitious composites are cast via the following general procedure: 1) dry mixing of constituents (cementitious materials: cement, fly ash, and silica fume, etc.; and fine coarse aggregates); followed by 2) wet mixing by introducing water and superplasticizer slowly to the dry constituents. The mixing process is modified depending on the reinforcement method. For example, for fiber reinforcement, the wet mixing stage is subdivided into three small stages: 1) wet mixing with a portion of the required water content; 2) addition of fibers; and 3) wet mixing with the remaining portion of the required water content.

In the case of bio-reinforced cementitious composites, the microbial strains can be incorporated as a suspension in the required water content at the wet-mixing stage. While nutrients, calcium source, and urea are introduced in the dry-mixing phase (Amieghemen et al. 2026; Bahja et al. 2025; Fang and Achal 2025; Priyadharsini et al. 2025; Wilson et al. 2025), this methodology severely impacts the survivability of the microbes due to the intense forces applied by the shear mixer. Particularly the rapid motion and collision of aggregates can damage the cellular walls of the microbes. To enhance the survivability of microbial strains during the mixing and casting process several studies have been conducted to utilize microbial strains that can sporulate (Booth and Jankovic 2022; Frahat et al. 2025; Sovljanski et al. 2025). In this process, microbial cultures are cultivated at the associated optimal conditions (pressure, temperature, and nutrients). The microbial cultures are incubated for several days and then centrifuged. Due to the incubation period, the nutrients are consumed, forcing the microbial culture to starve and sporulate. The centrifuging is applied to extract the spores (pellets) from the cultural medium (supernatant). The spores are added to the required water content and used during the wet-mixing phase.

Immobilization enhances survivability of microbial strains. Immobilization attaches microbes onto solid carriers to shield them from high pH, mixing shear forces while allowing for nutrient and ion transport needed for calcite precipitation (Chen et al. 2016; Gupta et al. 2018; Shen et al. 2021; Yang et al. 2022). Common carriers include expanded clay, metakaolin, wood ash, porous minerals, fibers, and waste-based materials. The most common immobilization techniques include absorption/adherence (Shen et al. 2021), vacuum impregnation (Feng et al. 2022a; Jiang et al. 2024), surface coating, and encapsulation (Feng et al. 2022b; Sandalci et al. 2021; Xue et al. 2018; Ying et al. 2023). Encapsulation is an immobilization technique that develops a physical barrier that shields bacteria from shearing forces. The microbial strains have been encapsulated in polymer microcapsules, hydrogel/alginate beads, inorganic/organic shells, lightweight/clay pellets, and freeze-dried spore formulations.

The injection process involves delivering a microbial suspension and cementitious solution into cracks, pores and fractures (Nagy and Kustermann 2023; Phillips et al. 2018; Yu et al. 2022). The injection of the microbial suspension can be applied through two mechanisms that are multi-phase injection and single-phase injection. In the multi-phase injection approach, the microbial suspension is initially introduced. The suspension is allowed to reside for an extended period to ensure that the microbial cells infiltrate the voids and pores. The cementitious solution containing nutrients and feedstock (typically urea and calcium source) is then injected to initiate the calcite precipitation process. The injections are applied in repeated cycles with a resting period to facilitate the infiltration of voids and sealing of cracks via calcite precipitation. In the single-phase, a cementitious solution with microbial suspensions is injected at once. To avoid premature calcite precipitation during the infiltration process into voids and cracks, inhibitors are added to temporarily suppress the MICP activity. The inhibitors are applied to reduce the pH and temperature of the cementitious solution. Once the solution infiltrates the soil and/or cementitious composites, the pH and temperature are neutralized and allow the initiation of the calcite precipitation to seal the cracks.

The immersion process includes submerging samples (soil or cementitious composites) in a solution that consists of microbial strains, nutrients, and feedstock (Jin et al. 2022; Liu et al. 2019a, b; Wen et al. 2018). The microbial solution infiltrates the samples through mass transfer by diffusion and convection to fill pores and cracks. Researchers have investigated reinforcing (pre-introducing) the samples with microbial strains prior to submerging in the feedstock/nutrient solution. The immersion process can be applied several times to re-seal the voids and cracks. Immersion can promote comparatively uniform precipitation in small laboratory specimens when solution access occurs from multiple surfaces. However, it cannot be assumed to maximize uniformity or yield across applications. The outcomes depend on specimen dimensions, pore connectivity, crack geometry, treatment cycles, and transport of cells, nutrients, carbonate species, and calcium ions. Its effectiveness therefore requires case-specific validation, particularly at larger scales. Finally, surface percolation is the application of microbial suspension with nutrients/feedstock by spraying the exterior surface of a sample (soil and cementitious composites) (Chen et al. 2023; Cheng and Cord-Ruwisch 2012). The solutions infiltrate the near surface cracks via capillary action, while deep voids are infiltrated through gravity.

## Applications

### Civil infrastructural and structural systems

**Fig. 4:**
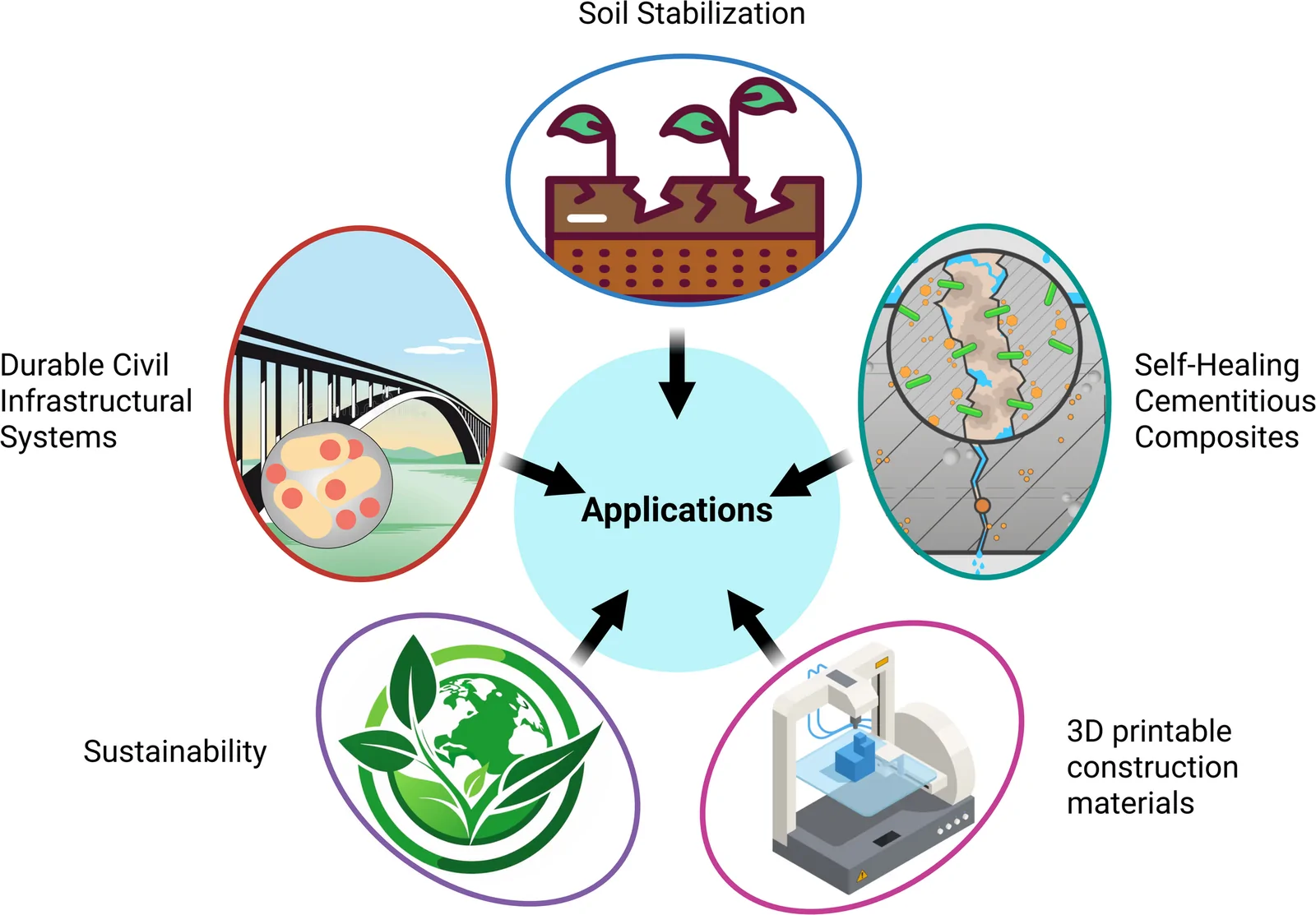
Bio-cementitious composites have diverse applications. Bio-cement enables self-healing cementitious composites, soil stabilization, enhanced durability of civil infrastructure, carbon sequestration for sustainability, and the development of 3D-printable construction materials. Created in https://BioRender.com

Fig 4. illustrates the diverse applications of bio-cement including for civil infrastructural and structural systems. Some microorganisms form dormant spores and tolerate prolonged exposure to harsh natural or engineered environments, including alkaline conditions (Bibi et al. 2018; Gowthaman et al. 2019; Huang et al. 2022a; Li et al. 2023; Nasser et al. 2022; Prajapati et al. 2023; Rahul Rollakanti and Srinivasu 2022; Yuan et al. 2026). However, persistence in non-cement environments does not demonstrate century-scale viability within cementitious matrices. Long-term survival and reactivation depend on the strain, delivery or encapsulation method, nutrient and moisture availability, pore structure, and exposure history, and evidence over multi-decade service periods remains limited. Microorganisms can detect environmental stressors and induce sporulation (creation of dormant bacterial cells with minimal metabolic activity). As such, the incorporation of calcite forming microbes in cement-based materials can significantly enhance the durability of structures such as wastewater treatment plants, structural foundation that is embedded deep in soil, and structural elements that are continuously immersed in deep sea/oceans (such as bridges, foundations for offshore oil rigs, and foundations for offshore wind turbines).

Initially, microbial strains have been included in soil along with nutrients and calcium sources to initiate MICP (Bibi et al. 2018; Gowthaman et al. 2019; Prajapati et al. 2023). The calcite precipitation increases the compressive strength and stiffness of the soil. Furthermore, the precipitated calcite reduces hydraulic conductivity and improves the resistance to erosion and liquefaction. As such, microbial strains have been utilized to stabilize slopes against rainfall-induced erosion, strengthening embankments and foundations, protecting coastal or dam structures, and improving seismic performance. Over the past decade, research has investigated the utilization of microbial strains to achieve self-healing of cementitious composites post a cracking event (Chang et al. 2024; Jia et al. 2025; Pungrasmi et al. 2019; Sohail et al. 2022). In general, once a cracking event occurs, water and oxygen infiltrate the material and provide the essential elements for initiating the MICP process. The calcite seals the cracks, reduces permeability, slows the reinforcement corrosion and extends the service life for civil infrastructures. It is worth noting that MICP has been used to immobilize heavy metals such as cadmium (Kang et al. 2014).

### Sustainability and carbon sequestration

The construction sector uses 30 billion tons of concrete per year with an increasing projection due to future demands (Ellis et al. 2020). Cement production (a binding agent for concrete) is an energy intensive process and generates 8% of the annual worldwide CO_2_ emissions (Malhotra, 1999). According to the 2016 Paris Agreement on climate change, there is a need to reduce carbon emissions of cement industries (Zhang et al. 2024). This section discusses potential applications to develop sustainable and carbon negative materials.

#### Sustainable substrates

MICP can be rendered more sustainable by substituting conventional, high‑purity chemical reagents with waste‑derived, low‑impact carbon sources and by directly utilizing CO_2_ and dissolved inorganic carbon (DIC). Diverse low‑cost organic substrates, including molasses and other sugar industry by‑products (Achal et al. 2013; Achal and Pan 2011), corn steep liquor (Achal et al. 2010; Joshi et al. 2018), as well as food industry and brewery/distillery effluents (Achal et al. 2009a; Meng et al. 2021; Pakbaz et al. 2020) have been shown to sustain ureolytic and other carbonate‑precipitating microorganisms while simultaneously valorizing agricultural and industrial residues. From the inorganic perspective, the use of bicarbonate‑/carbonate‑rich waters (DeJong et al. 2010; Seifan et al. 2017) and the integration of carbonic anhydrase producing microbes with flue gas or other CO_2_ streams facilitate in situ conversion of CO_2_ to carbonate, thereby promoting CaCO_3_ precipitation (Achal and Mukherjee 2015; Mwandira et al. 2023, 2024). When combined with carbonate‑rich or alkaline industrial by‑products (e.g., limestone fines, CaCO_3_ wastes, steel slags), these strategies can further improve resource efficiency and enhance CO_2_ sequestration in bio‑cemented soils and concretes (Mujah et al. 2017; Omoregie et al. 2023). Moreover, eco-conscious construction can be advanced by pairing green concrete**,** which incorporates recycled aggregates and industrial residues, with bio-cement, thereby lowering the overall environmental burden (Al-Sabaeei et al. 2022). Integrating bio-cement into such materials enhances their sustainability potential and supports the development of more environmentally responsible building systems. In a similar vein, industrial by-products like fly ash and slag can serve as effective substrates for bio-cement, opening pathways for future research to explore innovative recycling strategies that yield high-performance, low-impact construction materials (Liu et al. 2023b). Taken together, leveraging waste-derived substrates alongside CO_2_-based inputs presents a practical strategy to reduce the environmental footprint of bio-cement production, enhance carbon recycling, and lessen dependence on traditional cement materials.

#### Carbon negative materials

To develop carbon negative materials, several approaches have been utilized including 1) carbon curing (Dixit et al. 2021; Ravikumar et al. 2021; Zhang et al. 2017), 2) carbonation of aggregates and cementitious materials (Qiu 2020; Tang et al. 2023; von Greve-Dierfeld et al. 2020), 3) use of carbon capturing materials such as biochar (Fang et al. 2023; Liu et al. 2023), oyster shells (Kong et al. 2022), coral reefs (Mi et al. 2023), and nanomaterials, and 4) use of microbial strains. As discussed previously several calcite precipitation pathways utilize carbon dioxide including oxidation of organic salts, sulfate reducing bacteria, and cyanobacteria (Aghili et al. 2026). It is worth noting that photosynthetic bacteria such as cyanobacteria can promote long-term carbon storage through incorporation of fixed atmostpheric CO_2_ into calcite. MICP studies have reported mineralization efficiencies ranging from 20%–80% of supplied calcium under favorable conditions, converting biologically influenced inorganic carbon into stable carbonate phases (Stocks-Fischer et al. 1999). In addition, cyanobacterial biomass typically contains approximately 40%–50% carbon on a dry weight basis (Chisti 2007). Assuming complete conversion of inorganic carbon into biomass, this corresponds to approximately 1.5–1.8 kg CO_2_ fixed per kg dry biomass produced. These values highlight that the carbon-negative potential of photosynthetic construction materials depends not only on biological CO₂ uptake but also on the fraction of carbon retained within durable mineral or composite matrices. As such, cyanobacteria can be integrated into the manufacturing of cementitious composites to consume carbon dioxide from the surrounding environments (Lin et al. 2025).

It is worth noting that for material to be carbon-negative, carbon fixation must exceed the life-cycle greenhouse gas emissions. Net emissions should consider emissions from manufacturing processes (binders, feedstock, and enzymes), transportation, curing, maintenance, and end-of-life. Life-cycle assessments have illustrated context dependent reduction rather than universal negativity (Alotaibi et al. 2022; Deng et al. 2021). Accordingly, the strongest supportable conclusion is that selected designs may become net negative when low-clinker binders and waste inputs are combined with verified durable carbon storage and full balance remains below zero.

#### Petroleum engineering

Abandoned wells release methane and volatile organic compounds (VOCs) to the environment and surrounding soil leading to greenhouse gas effect, groundwater contamination and adverse impacts on public health (DiGiulio et al. 2023; Hachem and Kang 2023; Williams et al. 2021; Zhou et al. 2021). The US EPA recorded more than 3 million abandoned wells (DiGiulio et al. 2023). Concrete is used to seal those wells and encase walls of oil/gas wells. Concrete is porous and allows leakage of harmful materials. Novel projects use sealants that consist of bacteria with nutrients to block cracks/voids (Hiebert et al. 2022). A potential useful microbial strain is sulfate reducing bacteria that are anaerobic methanotrophic bacteria (Caesar et al. 2019; Seifan et al. 2016; Zhu and Dittrich 2016b).

### 3D Printable biomaterials for construction

In recent years, researchers have investigated the utilization of MICP to 3D printable biomaterials such as a layer of calcium carbonate (Erdmann et al. 2022b), small-scale spatial structures (less than 10 cm) (Antorveza Paez et al. 2025; Fredricks et al. 2021; Hirsch et al. 2023; Nething et al. 2020), and 3D printable molds for bio-cement bricks (Morrison et al. 2025). There are two broad 3D printing strategies that are: (1) direct bioprinting of MICP-active mixtures, and (2) indirect MICP treatment of 3D-printed profiles. In direct approaches, bacteria, nutrients, and calcium sources are co-extruded with a granular medium via nozzle-based deposition systems. The printed structures with the granular medium act as initiation sites for calcite precipitation. The indirect approach consists of the following steps: (1) 3D printing of molds, lattices, or scaffolds using polymers, gypsum, or cement-based; (2) placement of unconsolidated sand/soil; and (3) treatment with bacterial and cementation solutions that infiltrate the sand/soil medium.

However, the translation of MICP from retrofitting applications (soil stabilization and crack healing) to 3D printable construction systems introduces complex bottlenecks such as (1) control parameters that define materials’ rheology, buildability, and stability while ensuring biological viability and reaction kinetics; and (2) optimization of material distribution to ensure uniform mass-transportation and calcite precipitation.

## Challenges and limitations

Each microbial strain requires optimization of medium composition and nutrients to achieve a mechanical strength comparable to regular cementitious composites. Most research conducted to date is limited to early strength and self-healing performance. As such, the viability and functionality of the embedded microorganisms over decades have not been investigated, particularly in continuously changing environments (temperature, moisture, pH, and nutrient depletion). Preliminary life-cycle assessments illustrate absorption of carbon. However, these assessments are based on assumptions about raw materials, energy usage, transportation, service life, and end-of life. High-performance cementitious composites rely on significant cement content and energy-intensive processes. The net emissions might be reduced with the utilization of microbial strains but are not fully mitigated. Also, the research conducted was in controlled environments that provided optimal conditions including exposure to nutrients, moisture, and carbon dioxide. As such, the results obtained might not be realistic for open-air site conditions. Also, the rate of infiltration for carbonates, moisture, and nutrients is limited in dense matrices for large elements. This can restrict the CO_2_ sequestration. Another challenge is the scaling of bio-enhanced cementitious composites that require reliable, low-cost production of spores, nutrients, specialized admixtures, and tailored curing regimes. These processes are not yet optimized for the construction industry. Novel bio-enhanced and carbon-negative cementitious composites are expensive when compared to conventional concrete. As a result, developers and contractors are slowly adopting bio-enhanced composites due to the uncertainty associated with long-term behavior and supply. Furthermore, the long-term durability of bio-reinforced cementitious composites is under investigation with few research articles assessing feasibility beyond 120 days (Table 6). Finally, the codes and standards need to be revised to include procedures for fabricating, handling, and characterization of bio-enhanced cementitious materials, and design of elements. Further research is needed to standardize the life-cycle assessment protocols.

**Table 6:** Research for long-term durability bio-reinforced cementitious composites

Research	Matrix and microorganisms	Duration	Evidence type and reported observation
Bundur et al. (2017)	Mortar with unencapsulated vegetative *S. pasteurii*	330 days	*Direct Viability Evidence:* Viable bacteria recovered from hardened mortar, including cells identified in the vegetative state
Zou and Wang (2024)	Mortar with unencapsulated microorganisms	1,350 days	*Indirect Functional/Activity Evidence:* Microbial activity was reported after ~3.7 years, although later-age crack-healing efficiency was limited. Survival is inferred primarily from healing and mineralization
Xu and Sun (2026)	Concrete with microbial healing system	1 year	*Indirect Functional Evidence:* Exhibited crack closure and recovery of transport and mechanical properties
Javeed et al. (2025)	Mortar with co-cultures of *B. subtilis* and *S. pasteurii*	360 days	*Indirect Functional Evidence:* Evaluated later-age crack healing, water absorption and mechanical performance; healing of 0.3 mm cracks
Justo-Reinoso et al. (2022)	Cementitious composites with *B. cohnii* spores protected by air-entraining systems/carriers	9 Months	*Indirect Functional Evidence:* Successful healing of cracks formed at later ages indicates retained spore germination and mineralization capacity
Jiang et al. (2026)	Expanded-perlite immobilized *B. pasteurii* spores under simulated cementitious conditions	180 days	*Direct Survival and Activity Evidence:* Measured spore survival, urease activity and mineral production in simulated conditions
Teshome & Battula (2025)	Concrete with incorporated *B. subtilis* spores	150 days	*Direct Viability Evidence:* Viable cell counts fell below detection 120 days providing a limitation of direct bacterial incorporation
Jonkers et al. (2010)	Cement stones with alkali-resistant bacterial spores	135 days	*Direct Viability Evidence:* Viability declined below the detection limit

Direct viability evidence refers to recovery/culture or viable cell or spore measurements; direct activity evidence refers to measured metabolic or enzymatic activity; and indirect functional evidence refers to crack healing, property recovery, or mineralization without direct confirmation of viable organisms. These evidence types are not equivalent

## Future directions and innovative research

### Synthetic biology

The microbial mechanisms outlined in “Distinct contribution of this review” section can contribute to calcium carbonate formation either through cooperative interactions or as independent pathways. To optimize cementation performance, metabolic engineering efforts have largely converged on three principal directions: (1) optimization of urease activity (Hoffmann et al. 2021; Liang et al. 2018; Ma et al. 2020; Zhang et al. 2019); (2) strengthening microbial resilience to environmental stress (Dorfan et al. 2023; Kiledal et al. 2021; Liu et al. 2025); and (3) augmentation of CO_2_ conversion (Gilmour et al. 2024; Mamo and Mattiasson 2020; Preiss et al. 2015). These innovations have improved healing performance and long-term durability of conventional microbial self-healing systems (Rajadesingu et al. 2024; Wong et al. 2024). To engineer micro-organisms, whole genome sequencing needs to be conducted. For example, de novo sequencing of *S. pasteurii* BNCC 337394 and the complete 3.3-Mb chromosome of type DSM33 were conducted (Ma et al. 2020; Tuttle Matthew et al. 2024). Also, engineered carbonic anhydrase expressed in *B. subtilis* reduced CO_2_ from 3,800 to 820 ppm and produced calcite/vaterite in a controlled system, while environmental isolates have also been screened for carbonic anhydrase-mediated bio-cementation (Gilmour et al. 2024; Mwandira et al. 2024). The implementation of synthetic genetic circuits to precisely regulate biomineralization processes presents a promising avenue for exerting spatiotemporal control over cementation. Additionally, EPS are critical determinants of crystal nucleation, morphology, and accumulation. Leveraging synthetic biology in combination with omics-driven insights can enable the identification of key genetic determinants and the development of high-yield microbial strains tailored for enhanced biomineral production.

Despite these advances, the field still lacks fully integrated and systematic engineering frameworks due to several challenges. Environmental deployment requires careful evaluation of genetic stability, horizontal gene transfer with environmental bacteria, ecological interactions, and containment strategies (de Lorenzo 2018; Goold et al. 2018). Engineered strains may experience reduced fitness, genetic instability, or loss of engineered functions under fluctuating field conditions compared with controlled laboratory environments (Timmis et al. 2017). To address these challenges, approaches including biological containment systems, genetic safeguards, and engineered dependency circuits have been proposed to minimize unintended environmental impacts (Lee et al. 2018).

### Exploration of bacterial species and novel space explorations

Future advances in bio-cementation hinge on expanding the diversity of microbial systems beyond conventionally studied ureolytic bacteria such as *S. pasteurii*. Increasing attention is being directed toward non-ureolytic bacteria, including those that metabolize organic acids (Gowthaman et al. 2021), which can induce calcium carbonate precipitation while avoiding ammonia generation, thereby offering a more environmentally compatible alternative (Lin et al. 2022). In parallel, anaerobic bacteria present opportunities for bio-cement production in oxygen-limited settings, enabling applications in subsurface or underwater construction (Chuo et al. 2020). Beyond bacteria, fungal systems exhibit promise due to their ability to secrete organic acids and nucleate mineral precipitation, particularly in acidic or organic-rich environments where bacterial performance may be limited (Lin et al. 2023a). Additionally, microalgae contribute to carbonate formation through their photosynthetic and carbon-concentrating activity (Natsi and Koutsoukos 2022), making them attractive candidates for marine and coastal applications, including underwater infrastructure and reef restoration (Sasikumar et al. 2025). Collectively, these emerging biological platforms broaden the functional scope and environmental adaptability of bio-cementation technologies.

Space research offers a unique opportunity to develop bio-cement materials for extraterrestrial construction. Particularly, the U.S. Artemis Program aims to establish a sustained human base on the Moon that will enable future exploration of Mars. Unique microorganisms (e.g., extremophiles that can tolerate low gravity or radiation) can be engineered to convert locally available resources, such as regolith-derived minerals and organic waste, into biogenic materials and bio-cement. Such technologies could enable low-energy, self-healing, and locally manufactured construction materials, reducing the need to transport conventional cement and other heavy materials from Earth (Bell et al. 2026; Lee et al. 2025).

### Metabolic flux insights into microbial bio-cementation

Despite increasing interest in MICP for biocement and self-healing concrete, most studies remain focused on macroscopic outcomes such as urease activity, carbonate precipitation, cell viability, pH, etc. A system-level understanding of microbial metabolism within cementitious environments remains limited, with few studies applying transcriptomics, metabolomics, metabolic flux analysis (MFA), or genome-scale metabolic modeling (GEM). Recent reviews highlight that concrete represents a complex microbial habitat characterized by alkaline stress, osmotic stress, desiccation, nutrient limitation, restricted diffusion, and dynamic hydration, yet metabolic adaptation under these conditions remains poorly characterized (Povedano-Priego and Jroundi 2026). The emerging concept of living cement further emphasizes the need to engineer cementitious materials that support active microbial metabolism, requiring deeper understanding of microbial metabolic networks under physicochemical constraints (Luo and Li 2026). Although previous studies have elucidated key cellular processes in *S, pasteurii*, including ureolysis, nitrogen metabolism, ATP generation, membrane transport, calcium transport, and biomineral nucleation, they have largely lacked quantitative measurements of intracellular metabolic fluxes (Carter et al. 2023). Future research should therefore incorporate ^13^C metabolic flux analysis (Tang et al. 2009) to quantify carbon redistribution during MICP and reveal metabolic adaptations associated with energy production, stress responses, and biomineralization. Integration of ^13^C-MFA with transcriptomics, metabolomics, and metabolic models can enable predictive understanding of microbial behavior under varying cementitious conditions and accelerate the design of more robust self-healing concrete systems.

### Role of AI and machine learning

Large language models can mine the published research papers, reports, and standards for collecting the composition (mixture design) and performance of bio-enhanced cementitious composites. As a result, a large machine learning ready database can be generated to allow for AI-optimization of the cementitious composites (Li et al. 2025). Existing studies demonstrate the feasibility of data-driven bio-cement design, but most employ independent, project-specific datasets rather than a shared and interoperable database. For example, Huang et al. assembled 797 bacterial self-healing concrete observations from publications issued between 2000 and 2021 (Huang et al. 2022b). The database included 22 variables describing the bacterial system, healing mechanism, crack characteristics, exposure conditions, and healing duration. Similarly, Talamkhani compiled 402 unconfined-compression tests of MICP-treated sands using eight inputs (Talamkhani 2023). The eight inputs were the median particle size, uniformity coefficient, initial void ratio, calcium chloride concentration, urea concentration, urease activity, bacterial optical density, and calcite content. The study illustrated the predictive value of connecting microbial activity, treatment chemistry, soil characteristics, mineral production, and engineering performance. However, calcite content is generally measured after treatment. It is therefore useful for diagnostic prediction but cannot be treated as an initial design variable unless a separate model first predicts calcite formation. Erdmann et al. used a 20-run central composite experimental design to relate urea concentration, calcium chloride concentration, and bacterial suspension volume to the compressive strength of bio-cemented sand (Erdmann et al. 2022a). This example shows that database quality, systematic coverage of the design space, and experimental replication may be more important than data volume alone.

For cementitious self-healing systems, machine-learning models may predict crack-repair rates in concrete containing bacteria and polymeric fibers (Pessoa et al. 2024). Moreover, a bacteria-reinforced concrete database was developed to relate cement, fly ash, water to cement ratio, bacterial concentration, and coarse aggregate content to slump, flexural strength, and compressive strength (Onyelowe et al. 2024). Collectively, these studies demonstrate that AI can predict individual bio-cement outcomes. Nevertheless, separate models for crack closure, strength, or calcite formation do not yet provide an integrated platform for optimizing biological activity, engineering performance, environmental impact, and cost simultaneously. Thus, the applications demonstrated to date are project-specific predictive models for individual outcomes rather than integrated AI platforms for bio-cementitious materials.

By contrast, AI-integrated systems-biology and digital-twin platforms remain prospective research directions for bio-cementitious materials. These systems have not yet been validated as integrated design or monitoring systems. An additional opportunity lies in integrating AI with systems biology (transcriptomics, metabolomics, proteomics, and fluxomics) to identify complex regulatory relationships (Picard et al. 2021; Subramanian et al. 2020), which predict metabolic states, identify pathway bottlenecks, and improve the design of engineered microbial systems (Tarzi et al. 2024). For example, these approaches could enable prediction of optimal metabolic states for enhanced calcium carbonate biomineralization, identification of critical genes involved in mineral precipitation, and development of microbial strains with improved tolerance to alkaline and nutrient-limited cementitious environments. Furthermore, AI-driven digital twin frameworks combining biological data with material properties and environmental parameters could provide predictive simulations of microbial self-healing concrete performance under different structural and physicochemical conditions (Galal et al. 2026). Significant knowledge gaps remain in understanding microbial metabolism within cementitious environments. Addressing these gaps through quantitative metabolic analyses combined with AI-assisted modeling could establish a predictive systems biology framework to elucidate microbial metabolic reprogramming during calcium carbonate precipitation and guide the development of next-generation living cement materials in future.

## Conclusion and final remarks

MICP enhances the mechanical performance, durability, and functional behavior of cementitious materials while countering the impact of the construction industry on emissions. This review has described feasible pathways to achieve carbon-negativity and carbon-reduction through utilizing biologically enhanced cementitious composites. It also addresses the challenges associated with scalability of the bio-enhanced cementitious composites. The authors call for the standards and regulations to ensure consistent reporting of material development and characterization. A decision framework with four evaluation axes is proposed: (1) reaction effectiveness and mineral identity; (2) constructability and mass transport; (3) environmental burden, including nitrogen/sulfide byproducts and life-cycle carbon footprint; and (4) evidence of maturity from coupon-scale testing to field demonstration. This framework connects microbial and enzymatic mechanisms to immobilization, supplementary binders, crack-healing/durability outcomes, carbon accounting, and emerging manufacturing methods. In summary, bio-enhanced cementitious composites should not be considered as a direct replacement for conventional concrete materials as of today. However, bio-enhanced cementitious composites are an emerging platform technology that can augment existing materials. Researchers have illustrated the feasibility of integrating microbial strains for self-healing, soil-stabilization, and 3D printing on a micro/nanoscale. Further funding and research need to be conducted in the areas of long-term durability, availability, functionality (carbon sequestration), and life-cycle assessment. Looking ahead, AI is deriving the design of bio-enhanced cementitious composites and accelerating the optimization of the material’s composition and fabrication. To fully realize the potential of AI, substantial efforts are needed to build and standardize a machine learning-ready database linking material composition, environmental conditions, and performance.

## Data Availability

ChatGPT (OpenAI) was used to assist with language editing and to help identify potentially relevant references and knowledge nodes during manuscript preparation. All AI-generated suggestions were critically evaluated by the authors. The model and data are publicly available as indicated below: Code for Large Language Model (LLM): https://github.com/xiao-zhengyang/NEKO/. Input data for the LLM generate the knowledge graph: https://github.com/shikidj16/Biocement.
